# miR‐5188 augments glioma growth, migration and invasion through an SP1‐modulated FOXO1‐PI3K/AKT‐c‐JUN‐positive feedback circuit

**DOI:** 10.1111/jcmm.15794

**Published:** 2020-09-09

**Authors:** Renhui Yi, Shaochun Yang, Xian Lin, Liangying Zhong, Yuanyuan Liao, Zheng Hu, Tengyue Huang, Hao Long, Jie Lin, Zhiyong Wu, Cheng Xie, Shengfeng Ding, Jie Luo, Qisheng Luo, Ye Song

**Affiliations:** ^1^ Department of Neurosurgery Nanfang Hospital Southern Medical University Guangzhou China; ^2^ Department of Neurosurgery First Affiliated Hospital of Gannan Medical University Ganzhou China; ^3^ Department of Oncology Fujian Provincial Cancer Hospital The Affiliated Hospital of Fujian Medical University Fuzhou China; ^4^ Department of Laboratory Medicine The First Affiliated Hospital of Sun Yat‐sen University Guangzhou China; ^5^ Department of Ultrasonography First Affiliated Hospital of Gannan Medical University Ganzhou China; ^6^ Department of Neurosurgery Affiliated Hospital of Youjiang Medical University for Nationalities Baise China

**Keywords:** c‐JUN, FOXO1, glioma, miR‐5188, SP1

## Abstract

The biological effect and molecular mechanism of miR‐5188 have not been thoroughly investigated. The study aims at elucidating the role of miR‐5188 in glioma progression. Human glioma cell lines and tissues were used for functional and expression analysis. Cellular and molecular techniques were performed to explore the functions and mechanisms of miR‐5188 in glioma. In our investigation, we demonstrated that miR‐5188 promoted cell proliferation, the G1/S transition of the cell cycle, migration and invasion in glioma and reduced the lifespan of glioma‐bearing mice. miR‐5188 directly targeted FOXO1 and activated PI3K/AKT‐c‐JUN signalling, which enhanced miR‐5188 expression. Moreover, the c‐JUN transcription factor functionally bound to the miR‐5188 promoter region, forming the positive feedback loop. The feedback loop promoted glioma progression through activating the PI3K/AKT signalling, and this loop is augmented by the interaction between SP1 and c‐JUN. Moreover, it was also found that the miR‐5188/FOXO1 axis is facilitated by SP1‐activated PI3K/AKT/c‐JUN signalling. In glioma samples, miR‐5188 expression was found to be an unfavourable factor and was positively associated with the mRNA levels of SP1 and c‐JUN, whereas negatively associated with the mRNA levels of FOXO1. Our investigation demonstrates that miR‐5188 could function as a tumour promoter by directly targeting FOXO1 and participating in SP1‐mediated promotion of cell growth and tumorigenesis in glioma.

## 1 INTRODUCTION

Glioma, which is the most commonly diagnosed primary malignant neuroepithelial tumours and arises in the glial tissue, is characterized by an aggressive clinical phenotype.^1^, ^2^, ^3^ Although advances have been made in treatment modalities, including maximal surgical resection, chemotherapy and adjuvant radiotherapy, the extremely poor prognosis of patients with glioma has remained unchanged over the last three decades.^4^ Consequently, insights into the intricate molecular mechanisms underlying glioma progression may be useful to provide alternative strategies for the treatment of glioma.

Accumulating reports have supported that microRNAs (miRNAs) participated in numerous processes, including development, proliferation, cell cycle control and cellular differentiation, and are involved in regulating many human cancers.^5^, ^6^, ^7^, ^8^, ^9^, ^10^, ^11^ miRNAs may serve as tumour suppressors or oncogenes depending on the cancer type. Recently, many researchers demonstrated the involvement of miRNAs in the modulation of glioma cell growth, migration, invasion and recurrence.^12^, ^13^, ^14^ Recent studies suggested the powerful function of miR‐5188 in cancer progression.^15^, ^16^ However, the biological effects of miR‐5188 in glioma remain insufficiently understood.

In the current investigation, we identified the important role of miR‐5188 in glioma by examining miR‐5188 expression in glioma and non‐tumour brain specimens and the correlations between the expression of miR‐5188, SP1, c‐JUN and FOXO1. We discovered an atypical feedback loop involving miR‐5188‐FOXO1‐PI3K/AKT‐c‐JUN augmented by SP1. Furthermore, the identified loop promotes glioma cell growth, migration and invasion. Collectively, our data demonstrate a new mechanism through which miR‐5188 modulates glioma progression.

## 2 MATERIALS AND METHODS

### 2.1 Cell culture and collection of clinical tissues

The U251 and U87 cell lines were obtained from the Chinese Academy of Sciences (Shanghai, China). The glioma cells were cultured in Dulbecco's modified Eagle's medium containing 10% foetal serum purchased from Hyclone Corporation in a humidified atmosphere of 5% CO_2_ at 37°C as described previously.^17^ Two cohorts were adopted in this study. A total of 40 fresh primary human glioma samples and 17 fresh adjacent non‐tumour samples were obtained from the Nanfang Hospital, with the patients diagnosed with primary glioma without any treatment, from 2016 to 2017. Liquid nitrogen was applied for the storage of the fresh tissues prepared for further analysis. A total of 148 paraffin‐embedded glioma samples and 29 paraffin‐embedded adjacent non‐tumour samples were collected from Nanfang Hospital, with clinical and prognostic information from 2005 to 2010. All diagnoses were confirmed by pathology reports. The approved protocols were obtained from the Ethics Committee of the Nanfang Hospital, and prior consent was collected from the patients.

### 2.2 In situ hybridization

miR‐5188 expression levels in 148 paraffin‐embedded glioma and 29 adjacent non‐tumour samples were evaluated through in situ hybridization as previously described.^18^ Tissue sections were first dewaxed, rehydrated and subsequently treated with 3% H_2_O_2_. After incubation with proteinase K, the sections were then washed thoroughly. Pre‐hybridization was then performed, followed by hybridization with DIG‐labelled probes (Bis‐PR322) purchased from Biosense Bioscience Corporation. After washing and blocking, sections were subsequently incubated with anti‐DIG Fab fragments at room temperature for 1 h. The staining of miR‐5188 was finally conducted with a 3,3‐diaminobenzidine (DAB) substrate.

### 2.3 Lentivirus transfection

Lentiviral vectors possessing an hsa‐miR‐5188 precursor and their control sequence were purchased from GeneChem Corporation (Shanghai, China). The glioma cells were subjected to infection using the lentiviral vectors or their controls, and hsa‐miR‐5188 expression levels were confirmed in these cells.

### 2.4 Transfection of plasmid, siRNAs or miRNA inhibitors/mimics

The siRNA targeting FOXO1 and c‐JUN or mimics and inhibitors of hsa‐miR‐5188 were purchased from RiboBio Corporation (as shown in Table S1). c‐JUN, SP1 and FOXO1 plasmids were obtained from Vigene Biosciences Corporation. After seeding and growing, the glioma cells were subjected to transfection using siRNA, plasmid or miRNAs utilizing Lipofectamine TM 2000 (Invitrogen Biotechnology, China). After transfection, cells were collected for succeeding experiments at 48‐72 h.

### 2.5 Real‐time fluorescence quantitative PCR (RT‐qPCR)

TRIzol solution was used for total RNA extraction from cells and harvested tissues. Cycling conditions were set as previously mentioned,^19^ and the genes expression was normalized to ARF5 and U6. A melting curve confirmed the specificity of the amplification products. The sequences of all the primers are presented in Table S2.

### 2.6 Western blotting

Western blotting analyses were conducted as described previously.^20^ Antibodies against FOXO1, c‐JUN, pPI3K (Tyr458), PI3K, p‐AKT (Ser473), AKT, CCND1, CDK4, SP1, Vimentin, N‐cadherin and β‐actin are provided in Table S3. An enhanced chemiluminescence reagent purchased from Thermo Corporation was applied for recording the signals.

### 2.7 Immunofluorescence

Immunofluorescence was conducted as previously described.^21^ Briefly, glioma cells were seeded on coverslips and were subjected to fixation and permeabilization. Then, the cells were applied for incubation using primary anti‐c‐JUN and anti‐SP1 antibody, followed by incubation with a secondary antibody. The cells were costained with DAPI, and an ECLIPSE 80i fluorescence microscope was adopted to detect the expression and location of c‐JUN and SP1.

### 2.8 Immunohistochemical staining

After deparaffinization and rehydration, the paraffin‐embedded sections proceeded to antigen retrieval. Inactivation of endogenous peroxidase activity was performed using a peroxidase blocking reagent, and non‐specific antigens were blocked using the serum, followed by treatment with the antibody. Details of antibodies are presented in Table S3. After washing, sections were proceeded to treatment using an HRP‐labelled secondary antibody, followed by reaction with a DAB substrate. After counterstaining with haematoxylin solution, sections were prepared for observing under a microscope. The staining intensity was scored as described previously.^10^ The score lower than or equal to 6 indicated low expression, while the score higher than 6 indicated high expression.

### 2.9 MTT assay

An MTT assay was performed to examination cell proliferation as previously described.^22^ Glioma cells were seeded and allowed for growth. At 1, 2, 3 and 4 days after cell plating, the cells were incubated with MTT (5 mg/mL) purchased from Sigma Corporation. The formazan crystals were solubilized in DMSO purchased from Sigma Corporation, and absorbance values (OD values) were then detected and recorded.

### 2.10 Colony formation assay

Glioma cells were seeded into 6‐well plates, with each well consisted of 100 cells. Fourteen days after incubation, colonies were rinsed with phosphate‐buffered saline (PBS) and were subjected to staining using haematoxylin. Colonies number in each well was counted under a microscope.

### 2.11 Cell cycle analyses and EdU incorporation assays

The experiments were conducted as described previously.^23^ For cell cycle analyses, glioma cells were collected after culture for 48 h, followed by a wash with cold PBS and fixing with 70% ethanol. The fixed cells were then subjected to incubation using propidium iodide (10 mg/mL) and RNase A (0.5 mg/mL). After wash, the labelled cells were analysed with flow cytometry. For the EdU incorporation assay, glioma cells were treated with the Apollo 488 In Vitro Imaging Kit purchased from RiboBio Corporation. The number of Edu‐positive cells was calculated using a microscope.

### 2.12 In vivo tumorigenesis in nude mice

An in vivo study was conducted to evaluate tumorigenesis as described previously.^24^ The 4‐ to 5‐week‐old female BALB/c‐nu mice for each group were adopted in the study and were routinely maintained in a barrier facility. U87 cells stably expressing miR‐5188 or negative control cells were subcutaneously inoculated into the flank of the mice (n = 5 per group). After inoculation, the mice were killed at day 25, and tumour xenografts were collected for further analysis. The tumour volume was calculated as maximum diameter × minimal diameter^2^/2. Survival curves were plotted, and Kaplan‐Meier analyses were performed in an intracranial glioma tumour model (n = 10 per group). The survival time was calculated as previously described.^25^ The approved protocols were obtained from the Ethics Committee of Southern Medical University.

### 2.13 Cell migration and invasion assays

In vitro cell migration and invasion assays were performed according to our previous study.^14^ For the cell migration assay, a total of 1 × 10^4^ cells in 100 μL DMEM medium without FBS were seeded on a Transwell apparatus (Costar, MA). In the lower chamber, 500 μL DMEM with 10% FBS was added as a chemoattractant. After the cells were incubated for 6 hours at 37°C in a 5% CO_2_ atmosphere, the insert was washed with PBS, and cells on the top surface of the insert were removed with a cotton swab. Cells adhering to the lower surface were fixed with methanol, stained with crystal violet solution and counted under a microscope in five predetermined fields (200×). All assays were independently repeated at least thrice. For the cell invasion assay, the procedure was similar to the cell migration assay, except that the transwell membranes were pre‐coated with 24 μg/μL Matrigel (R&D Systems, USA) and the cells were incubated for 8 hours at 37°C in a 5% CO_2_ atmosphere. Cells adhering to the lower surface were counted the same way as the cell migration assay.

### 2.14 Chromatin immunoprecipitation assay (ChIP)

Bioinformatics analyses revealed c‐JUN‐binding sequences inside the promoter region of miR‐5188. To prove this, chromatin was first enriched from U87 and U251 cells using the ChIP Kit purchased from Millipore Corporation. The immunoprecipitation was conducted with anti‐c‐JUN or IgG antibodies. IgG was applied as a negative control. Finally, RT‐qPCR and RT‐PCR analyses were conducted to evaluate the miR‐5188 promoter region enrichment with specific primers.

### 2.15 Co‐immunoprecipitation (Co‐IP)

Co‐IP analyses were conducted with a Pierce Co‐Immunoprecipitation kit purchased from Thermo Corporation as previously described.^26^ Briefly, proteins were collected from glioma cells and were subjected to protein quantification. Proteins in supernatant were then incubated with specific antibodies overnight. After elution with sample buffer, the proteins were denatured and were subsequently subjected to Western blotting analysis.

### 2.16 Luciferase reporter assay

TargetScan and miRWalk algorithm software predicted FOXO1 as a target gene of miR‐5188. A part of the FOXO1 3′UTR (wild‐type 3′UTR) was amplified and was then inserted into psiCHECK‐2 vectors. GeneTailor Site‐Directed Mutagenesis System purchased from Invitrogen Corporation was applied to generate the mutations of FOXO1 3′UTR targeted by miR‐5188 seed sequence. For reporter assays, miR‐5188 mimics or inhibitors were cotransfected into U87 cells with the established vectors. The Dual‐Luciferase Reporter Assay System purchased from Promega Corporation was applied for the detection of luciferase activity.

### 2.17 RNA immunoprecipitation (RIP) assay

RNA immunoprecipitation assays were examined using a RIP assay kit (Millipore Corp, Billerica, MA, USA). According to the manufacturer's protocol, the protein‐RNA complex was isolated, and anti‐AGO2 or IgG was added to the reaction system for immunoprecipitation. After RNA purification, the immunoprecipitated RNA was subjected to QPCR and/or PCR. IgG served as a negative control.

### 2.18 Statistical analysis

All data analyses were conducted with GraphPad Prism V7.0 or IBM SPSS V22.0 software. Data are presented as mean ± SD. Student's *t* tests were applied for comparisons between two groups, and one‐way ANOVA analyses were applied for comparisons between multiple groups. A parametric generalized linear model with random effects was adopted for analysis of tumour growth and the MTT assay. Spearman's correlation coefficient was applied to elucidate the correlations between miR‐5188 and FOXO1 or miR‐5188 and SP1 or miR‐5188 and c‐JUN. Kaplan‐Meier method and log‐rank test were adopted for survival analysis. All statistical analyses were two‐sided, and statistical significance was indicated as: *P < .05; **P < .01; ***P < .001.

## 3 RESULTS

### 3.1 miR‐5188 augments glioma cell proliferation, migration and invasion

**FIGURE 1:**
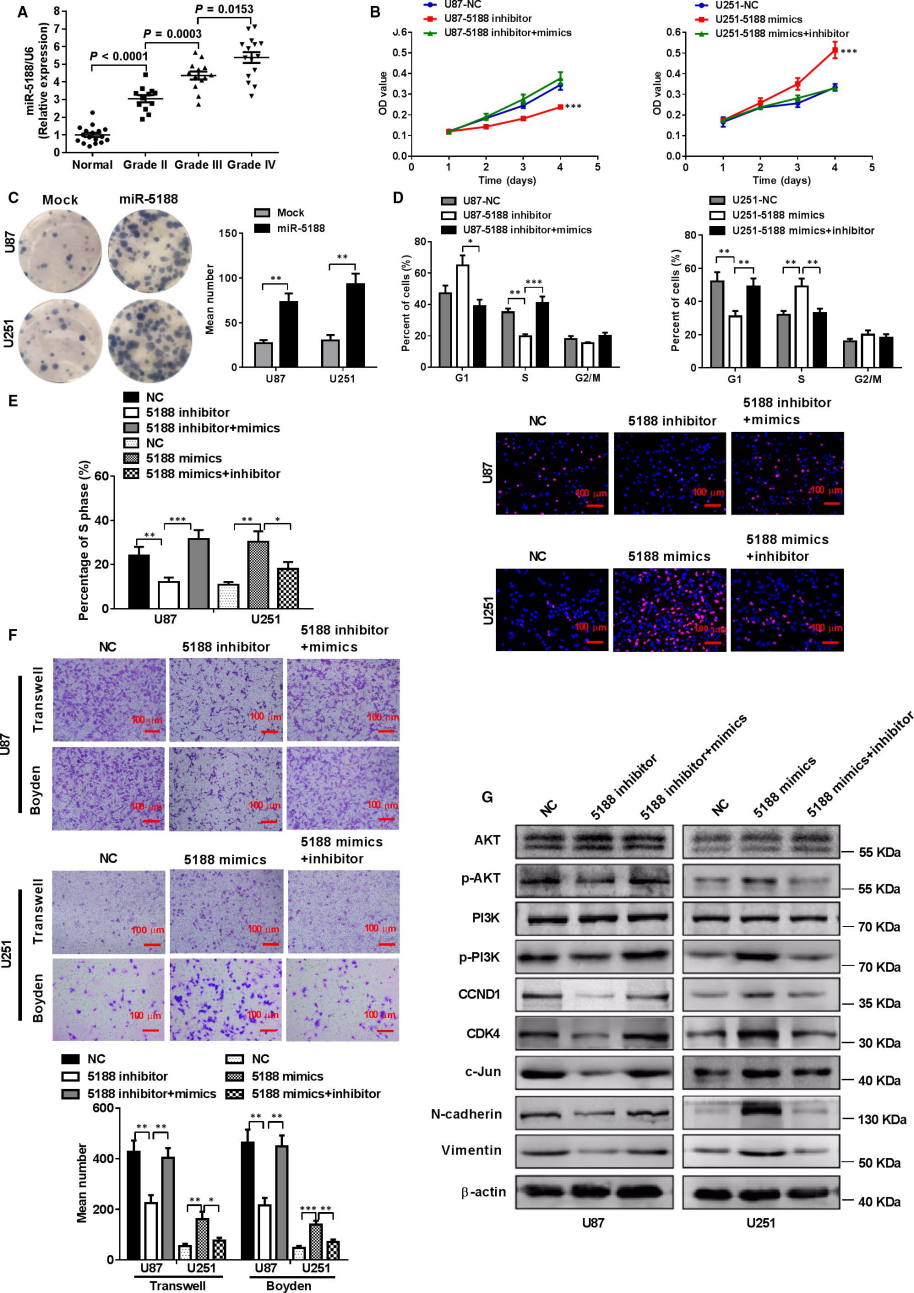
miR‐5188 is elevated in glioma tissues and promotes glioma cell proliferation, migration and invasion in vitro by up‐regulating PI3K/AKT activity. A, Increased levels of miR‐5188, as assessed using real‐time quantitative PCR (RT‐qPCR), were positively correlated with the pathology classification status. An unpaired t test was applied for assessing the results of this assay in normal tissues (n = 17), grade II glioma (n = 12), grade III glioma (n = 13) and grade IV glioma (n = 15). An MTT assay (B), colony formation assay (C), cell cycle analysis (D) and EdU incorporation assay (E) were applied to investigate the impact of miR‐5188 on cell proliferation in U87 and U251. Scale bars, 100 μm. F, A transwell assay and Boyden chamber assays were applied to elucidate the impact of miR‐5188 on cell migration and invasion in U87 and U251. Scale bars, 100 μm. G, Western blotting analyses were applied to assess P‐PI3K, PI3K, P‐AKT, AKT, CDK4, CCND1, c‐JUN, N‐cadherin and vimentin protein levels in U87 and U251 cells treated with miR‐5188 inhibitor or mimics. *P < .05; **P < .01; ***P < .001

To figure out the biological effect of miR‐5188 in glioma progression, we first investigated miR‐5188 levels in fresh non‐tumour tissues and fresh glioma tissues through RT‐qPCR. miR‐5188 was overexpressed in glioma tissues but weakly expressed in healthy brain tissues, and elevated miR‐5188 levels were positively associated with tumour grade (Figure 1A).

To further investigate the function of miR‐5188 in glioma cells, miR‐5188 mimics, inhibitors or lentiviral vectors were transfected into U87 or U251 cells, respectively. The increased levels of miR‐5188 were detected in miR‐5188 mimic‐ and lentiviral vector‐infected glioma cells compared with the counterpart, as assessed using qRT‐PCR (Figure S1A‐C). Subsequently, the effects of miR‐5188 on glioma progression were examined in vitro. MTT assays (Figure 1B), colony formation assays (Figure 1C), cell cycle analyses (Figure 1D) and Edu incorporation assays (Figure 1E) showed that miR‐5188 overexpression markedly promoted cell proliferation and the G1/S transition of the cell cycle in U251 cells. Transwell and Boyden assays indicated that the miR‐5188‐overexpressed group exhibited promoted cell migration and invasion in comparison with the control group (Figure 1F). However, knockdown of miR‐5188 in U87 cells induced the opposite results.

While determining the mechanisms that miR‐5188 promotes glioma progression, we confirmed that overexpression of miR‐5188 up‐regulated protein levels of CDK4, CCND1, c‐JUN, N‐cadherin and vimentin. Interestingly, miR‐5188 inhibitor could restore the levels of cell cycle and epithelial‐mesenchymal transition associated proteins. Moreover, we applied Western blot to identify the most prominent signalling regulated by miR‐5188 and found that p‐PI3K and p‐AKT expression levels were increased in U251 cells with miR‐5188 up‐regulation. However, the expression pattern was attenuated in miR‐5188‐overexpressing U251 cells treated with miR‐5188 inhibitor (Figure 1G), While overexpression of miR‐5188 could reverse the inhibitory effect of miR‐5188 knockdown in U87 cells. Our findings reveal that miR‐5188 augments the activity of the PI3K/AKT signalling pathway, an important pathway for cell proliferation and metastasis.

### 3.2 miR‐5188 promotes tumorigenicity of glioma cells in vivo

**FIGURE 2:**
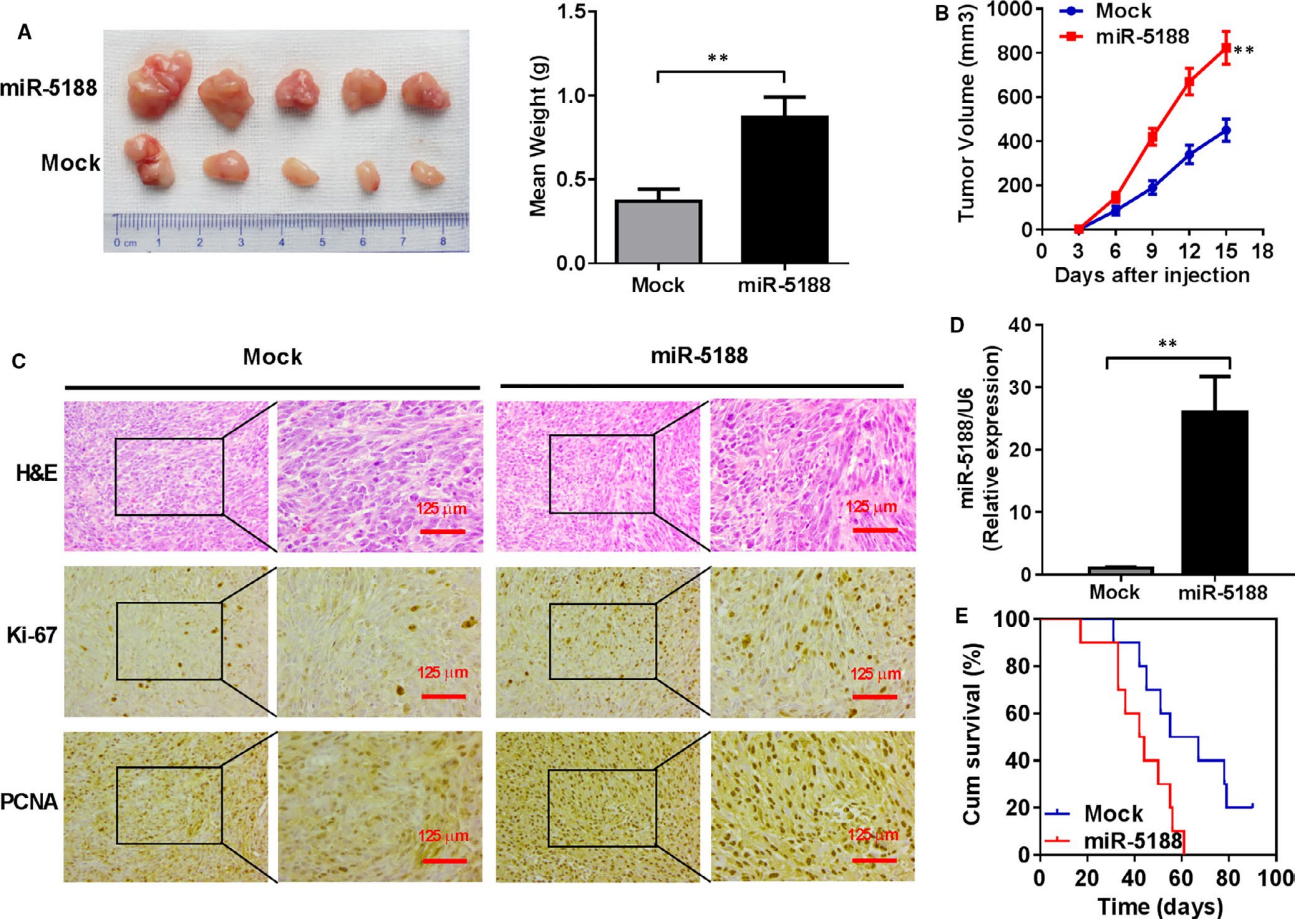
miR‐5188 augments glioma cell growth in vivo. A, Tumorigenicity of U87 cells was increased in miR‐5188‐overexpressing group in comparison with the control group. B, The growth curves of the tumours in nude mice were plotted to explore the impact of miR‐5188 on glioma growth. C, Representative images showing the H&E staining of primary glioma tissues and immunohistochemical analysis of Ki67 and PCNA expression. Scale bars, 125 μm.. D, RT‐qPCR analyses were applied to measure miR‐5188 expression levels in xenografts. E, Survival times calculated through a Kaplan‐Meier analysis revealed that miR‐5188 overexpression (miR‐5188) reduced survival time compared with untreated controls (Mock). **P < .01

In vivo tumorigenesis assays were applied to illustrate the effect of miR‐5188 on glioma cell proliferation by injecting stable miR‐5188‐overexpressing U87 cells or negative control cells into the nude mice. The average tumour weights for the miR‐5188 group and the mock group were 0.8692 and 0.375 g, respectively (Figure 2A), and the mice had higher tumour growth rates after miR‐5188 overexpression (Figure 2B). Immunohistochemical staining suggested the increased protein levels of Ki67 and PCNA in the miR‐5188 overexpressing group (Figure 2C). The RT‐qPCR analysis confirmed elevated miR‐5188 expression in the miR‐5188 overexpression xenografts in comparison with untreated control xenografts (Figure 2D). Survival analyses were conducted and elucidated that miR‐5188 overexpression (miR‐5188) led to a significantly shorter lifespan compared with untreated controls (Mock) (Figure 2E). Our findings reveal that miR‐5188 exerts a promotive effect on the tumorigenesis of glioma cells in vivo.

### 3.3 miR‐5188 suppresses FOXO1 through directly targeting FOXO1

**FIGURE 3:**
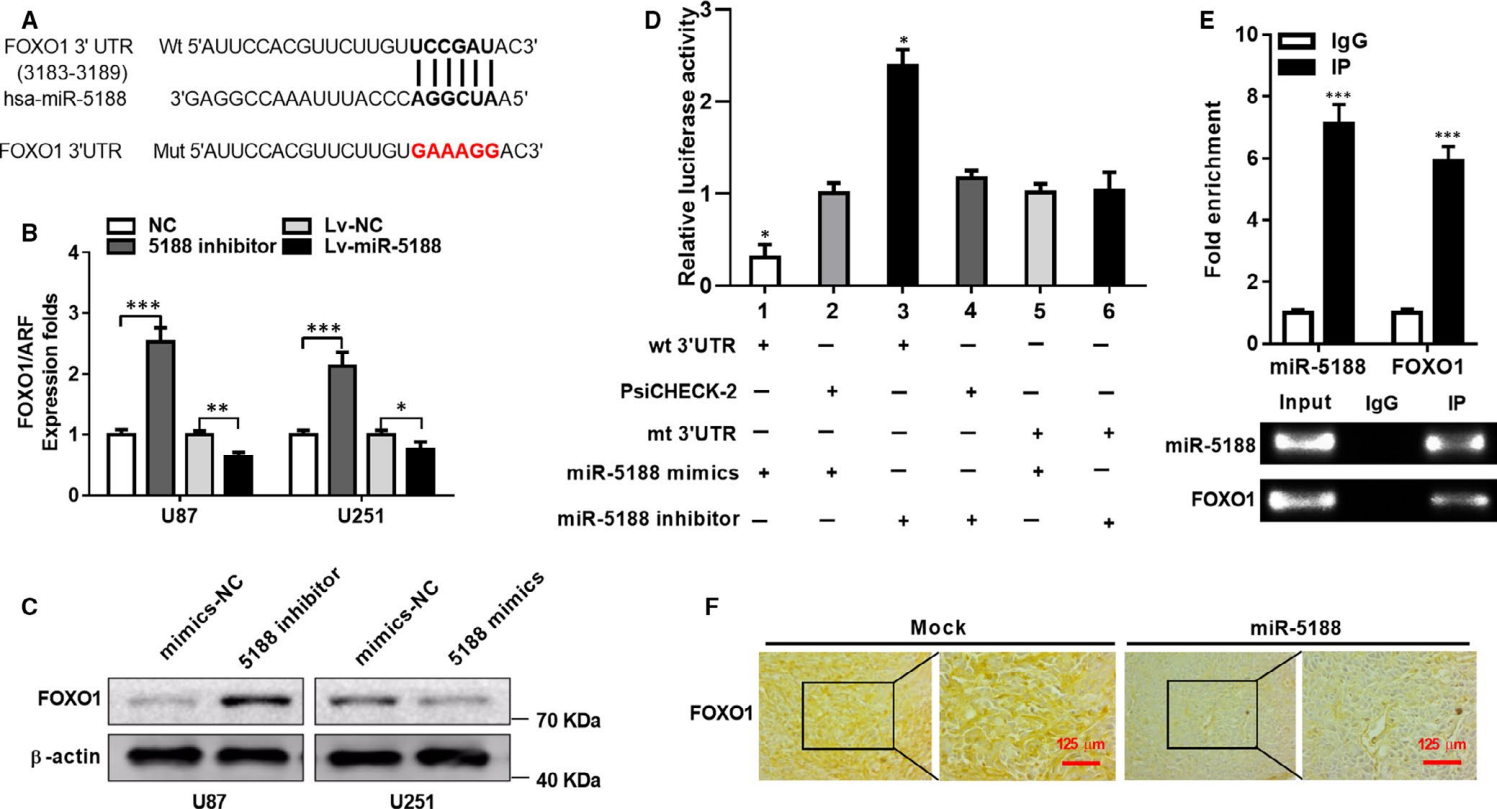
miR‐5188 down‐regulates FOXO1 through directly targeting FOXO1. A, miR‐5188 and its binding sites in FOXO1 3′UTR. A mutation was constructed in the sequences targeted by miR‐5188 seed region. B, FOXO1 mRNA levels were measured through RT‐qPCR in miR‐5188‐silenced U87 and miR‐5188‐overexpressing U251 cells and normalized to ARF5. C, Western blotting analyses were applied to investigate FOXO1 protein levels in miR‐5188‐silenced U87 and miR‐5188‐overexpressing U251 cells. D, A luciferase reporter assays were applied to elucidate the direct targeting of FOXO1 3′UTR by miR‐5188. E, RNA immunoprecipitation was conducted to confirm the interaction between AGO2‐bound miR‐5188 and FOXO1 mRNA. F, FOXO1 protein levels were measured through immunohistochemistry in xenografts. Scale bars, 125 μm. *P < .05; **P < .01; ***P < .001

TargetScan and miRWalk algorithms predicted FOXO1 as a direct target gene of miR‐5188 (Figure 3A). RT‐qPCR and Western blotting analyses verified that overexpression of miR‐5188 down‐regulated mRNA and protein levels of FOXO1 in U251 cells, while knockdown of miR‐5188 in U87 cells presented the opposite effects (Figure 3B,C). Transfection with miR‐5188 mimics impaired FOXO1 luciferase reporter activity (Figure 3D, as shown in lanes 1 and 2). However, the introduction of a miR‐5188 inhibitor exhibited the opposite effects (Figure 3D, as shown in lanes 3 and 4). The influence on luciferase activity was terminated under cotransfection using a mutated FOXO1 reporter (Figure 3D, as shown in lanes 5 and 6). In addition, RNA immunoprecipitation further confirmed the interaction between AGO2‐bound miR‐5188 and FOXO1 mRNA (Figure 3E). Moreover, immunohistochemistry of xenografts in the miR‐5188 group revealed a marked reduction in FOXO1 expression (Figure 3F). Collectively, these data demonstrate that miR‐5188 directly targets FOXO1 in glioma.

### 3.4 FOXO1 mediates the promotive effects of miR‐5188

**FIGURE 4:**
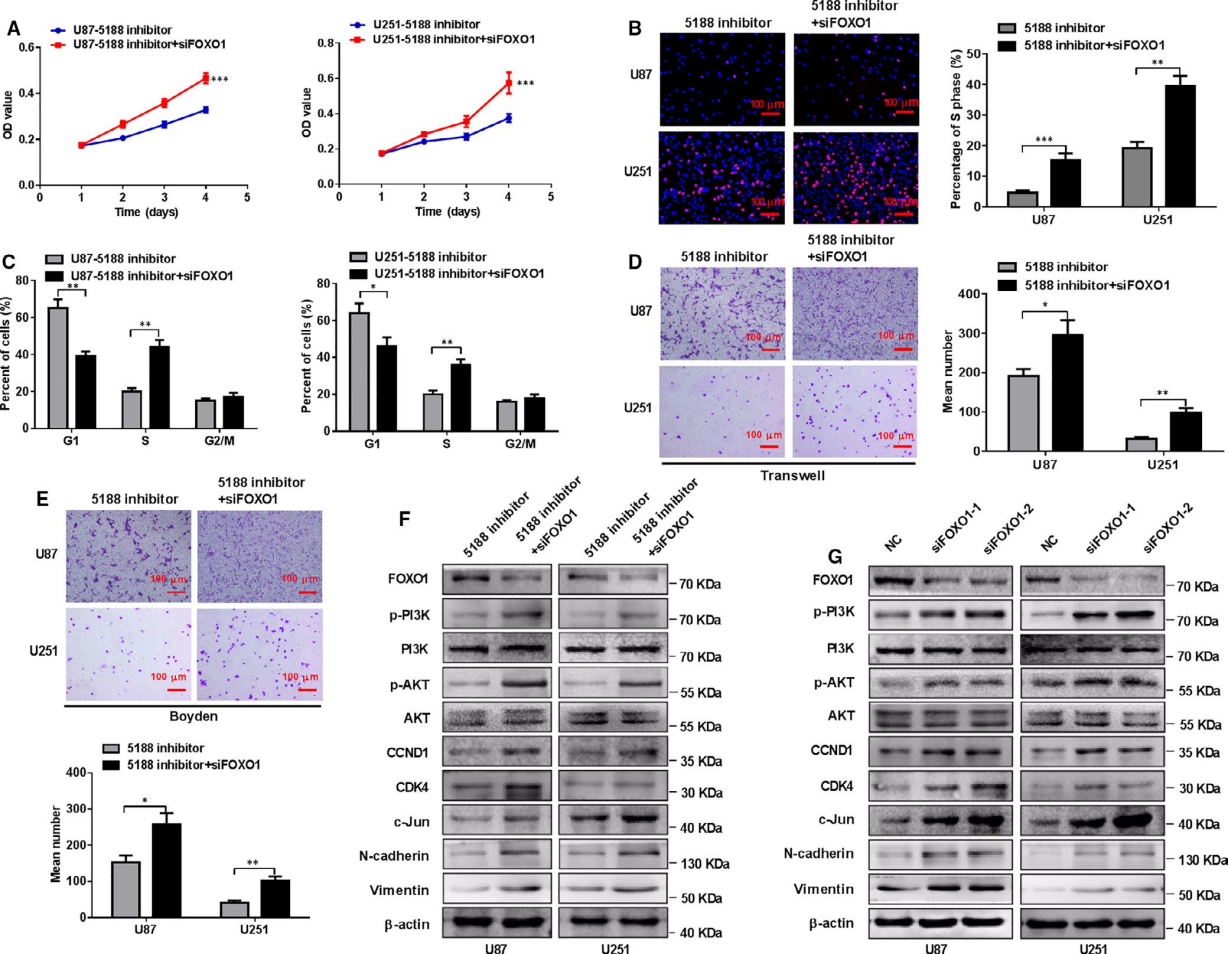
Silence of FOXO1 mitigates the inhibitory effects of miR‐5188 knockdown on glioma cell proliferation, invasion and migration. MTT assays (A), EdU incorporation assays (B), flow cytometry assays (C), transwell assays (D) and Boyden assays (E) were performed in U251 or U87 cells treated with control sequence, FOXO1 siRNA or miR‐5188 inhibitor, as indicated. Scale bars, 100 μm. F, Western blotting analyses were applied to measure the protein levels of FOXO1, P‐PI3K, PI3K, P‐AKT, AKT, CCND1, CDK4, c‐JUN, N‐cadherin and vimentin in U251 or U87 cells treated with control sequence, FOXO1 siRNA or miR‐5188 inhibitor, as indicated. G, Western blotting analysis analyses were applied to measure the protein levels of FOXO1, P‐PI3K, PI3K, P‐AKT, AKT, CCND1, CDK4, c‐JUN, N‐cadherin and vimentin protein levels in U87 and U251 cells transfected with control sequence or FOXO1 siRNA. *P < .05; **P < .01; ***P < .001

FOXO1 knockdown led to augmented cell proliferation measured by MTT and EdU incorporation assays and promoted the transition of G1 to S cell cycle in miR‐5188‐silenced glioma cells (Figure 4A‐C) (Figure S2A). Further experiments demonstrated that similar changes were observed in cell migration and invasion (Figure 4D,E). Furthermore, Western blotting analyses showed that FOXO1 knockdown enhanced the protein levels of P‐PI3K, P‐AKT, CCND1, CDK4, c‐JUN, N‐cadherin and vimentin in miR‐5188‐silenced U87 and U251 cells (Figure 4F), indicating that silence of FOXO1 could overcome the inhibition of glioma cell proliferation, invasion and migration mediated by miR‐5188 knockdown. Besides, FOXO1 knockdown elevated protein levels of P‐PI3K, P‐AKT, CDK4, CCND1, c‐JUN, N‐cadherin and vimentin in glioma cells (Figure 4G). Taken together, the obtained results confirm FOXO1 as a direct target gene of miR‐5188 and a contributor to miR‐5188‐mediated cell proliferation, invasion and migration.

### 3.5 c‐JUN binds to the promoter region of miR‐5188 to promote its transcription

**FIGURE 5:**
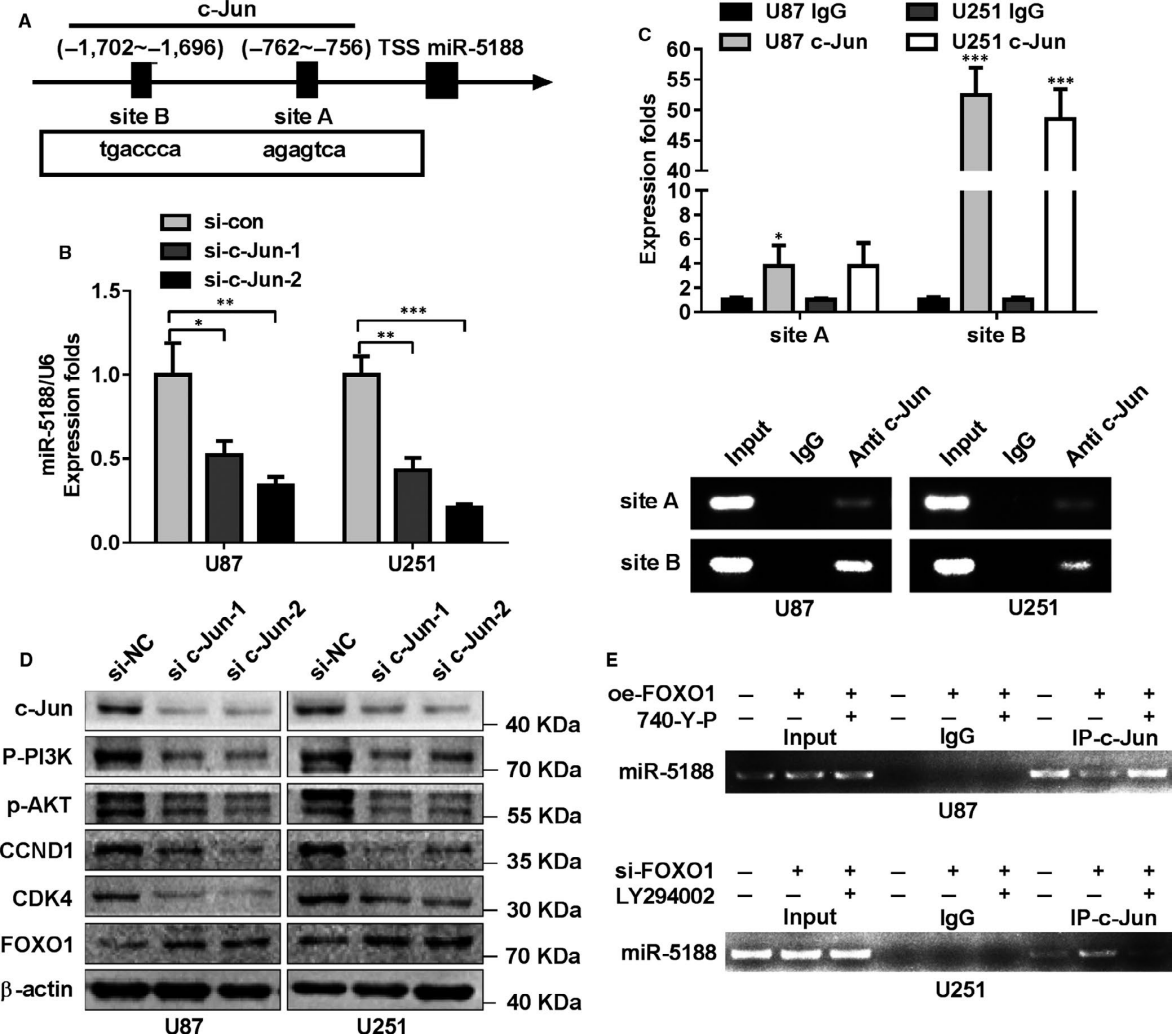
c‐JUN positively enhances miR‐5188 transcription by binding to its promoter region. A, Schematic diagram of the c‐JUN binding sites (site A and site B) inside miR‐5188 promoter regions. B, miR‐5188 levels were measured through RT‐qPCR in c‐JUN‐silenced U87 or U251 cells. C, RT‐qPCR and RT‐PCR analyses were applied to amplify c‐JUN‐binding sites A and B enriched from Chromatin immunoprecipitation assay (ChIP) using anti‐c‐JUN. D, Western blotting analysis analyses were applied to measure the protein levels of c‐JUN, P‐PI3K, P‐AKT, CDK4, CCND1 and FOXO1 expression after c‐JUN suppression. E, ChIP assays were conducted to investigate the impact of FOXO1 and PI3K signalling on c‐JUN‐augmented miR‐5188 transcription in glioma cells. *P < .05; **P < .01; ***P < .001

The ALGGEN (http://alggen.lsi.upc.es) and JASPAR (http://jaspar.genereg.net) databases were applied for analysing potential transcriptional factors binding to the promoter of miR‐5188 to examine the underlying regulation of miR‐5188 expression. Two putative c‐JUN‐binding sites were searched within the promoter region of miR‐5188 (Figure 5A). To illustrate the potential function of c‐JUN in the modulation of miR‐5188, we applied siRNAs to silence c‐JUN expression levels in U87 and U251 cells (Figure S2B). Then, RT‐qPCR confirmed the decreased miR‐5188 expression in glioma cells under c‐JUN knockdown (Figure 5B), suggesting c‐JUN as an upstream mediator of miR‐5188.

ChIP assays were subsequently performed to determine the endogenous binding with the c‐JUN antibody. As expected, the specific regions were enriched from immunoprecipitated chromatin DNA in comparison with the counterpart (Figure 5C). Moreover, P‐PI3K, P‐AKT, CCND1 and CDK4 protein levels were down‐regulated, while FOXO1 protein levels were up‐regulated after c‐JUN knockdown (Figure 5D). Above‐mentioned data indicated that c‐JUN could transcriptionally enhance miR‐5188 transcription. Intriguingly, we confirmed that PI3K signalling mediated the inhibitory impact of FOXO1 on miR‐5188 transcription stimulated by c‐JUN (Figure 5E). Collectively, the obtained data demonstrate that c‐JUN directly enhances miR‐5188 transcription, and there may exist a positive feedback circuit miR‐5188‐FOXO1‐PI3K/AKT‐c‐JUN in glioma.

### 3.6 SP1 interacts with c‐JUN to facilitate miR‐5188 expression in glioma

**FIGURE 6:**
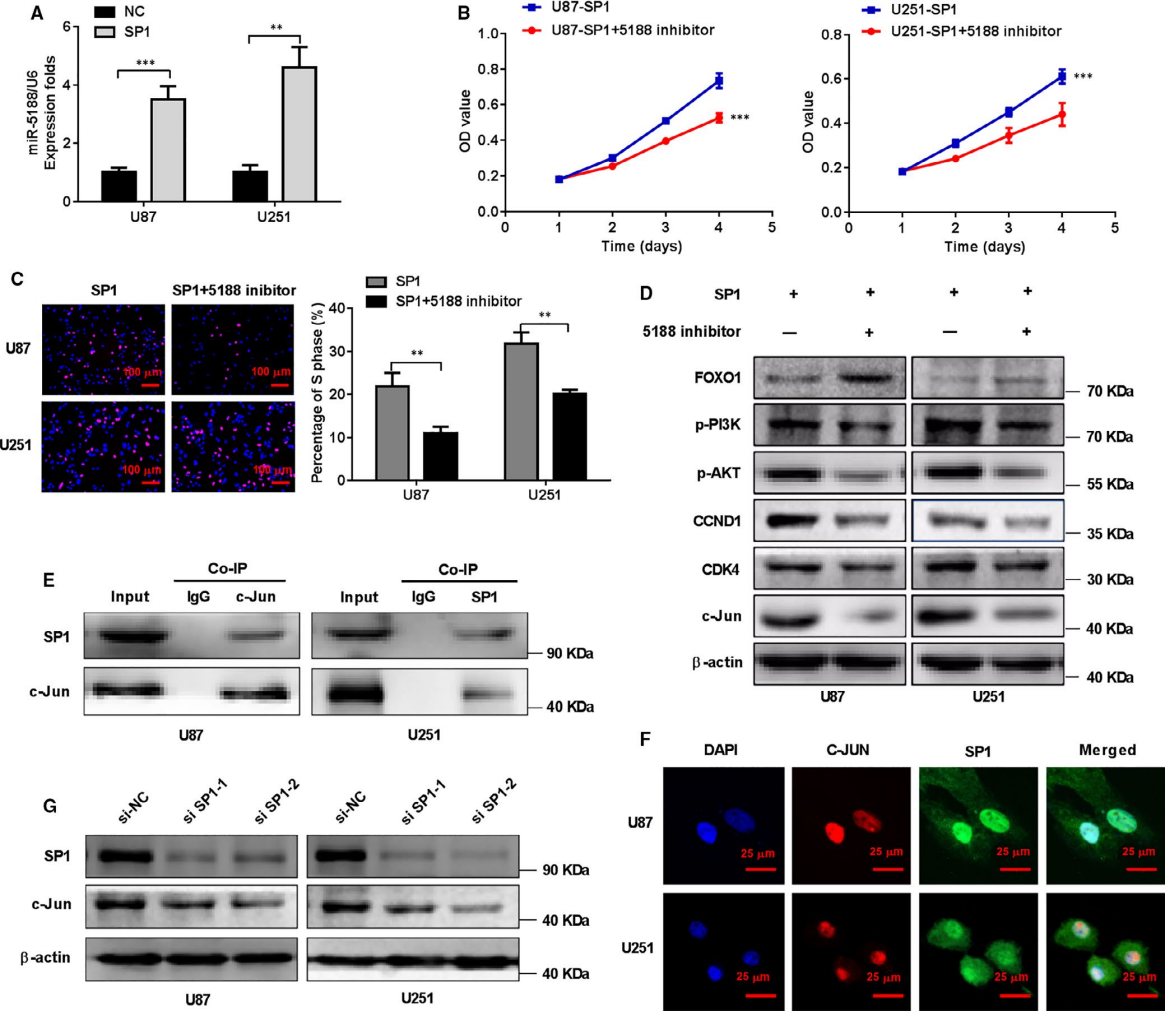
SP1 facilitates miR‐5188 expression in glioma. A, miR‐5188 levels were assessed through RT‐qPCR in U87 or U251 cells transfected with the control vector or SP1 plasmid, as indicated. MTT assays (B) and EdU incorporation assays (C) were performed in U87 and U251 cells treated with SP1 plasmid or both SP1 plasmid and miR‐5188 inhibitor. Scale bars, 100 μm. D, Western blotting analysis analyses were applied to measure the protein levels of P‐PI3K, P‐AKT, CCND1, CDK4, c‐JUN and FOXO1 in U87 and U251 cells treated with SP1 plasmid and miR‐5188 inhibitor. E, Co‐immunoprecipitation (Co‐IP) was applied for detecting the interaction between endogenous SP1 and c‐JUN in U87 and U251 cells. F, Immunofluorescence staining was applied to assess the nuclear colocalization of SP1 and c‐JUN protein in U87 and U251 cells. Scale bars, 25 μm. G, Western blotting analyses were applied to measure the protein levels of c‐JUN in U87 and U251 cells with SP1 knockdown. **P < .01; ***P < .001

During speculation of the specific molecular mechanisms of miR‐5188 and relevant association with c‐JUN, SP1 was considered as a crucial positive modulator of miR‐5188 (Figure 6A). A consecutive miR‐5188 knockdown could overcome the stimulatory effects of SP1 overexpression in glioma cells, as measured using MTT and Edu assays (Figure 6B,C). Besides, Western blotting revealed that miR‐5188 inhibition led to reduced protein levels of P‐PI3K, P‐AKT, CCND1, CDK4 and c‐JUN, but increased protein levels of FOXO1 in glioma cells with SP1 overexpression (Figure 6D), suggesting that miR‐5188 was enhanced by SP1 through the PI3K/AKT pathway. The endogenous Co‐IP assay showed the interaction between SP1 and c‐JUN in glioma cells (Figure 6E). Immunofluorescence showed the nuclear colocalization of SP1 and c‐JUN proteins in glioma cells (Figure 6F). Furthermore, we also found that levels of c‐JUN were decreased after SP1 knockdown through Western blotting in glioma cells, suggesting the regulation of SP1 on PI3K/AKT‐c‐JUN pathway (Figure 6G).

In conclusion, our findings suggest the induction of miR‐5188 expression by SP1 via the PI3K/AKT/c‐JUN signalling pathway.

### 3.7 The correlations between pathoclinical characteristics, miR‐5188 expression and its associated genes

**FIGURE 7:**
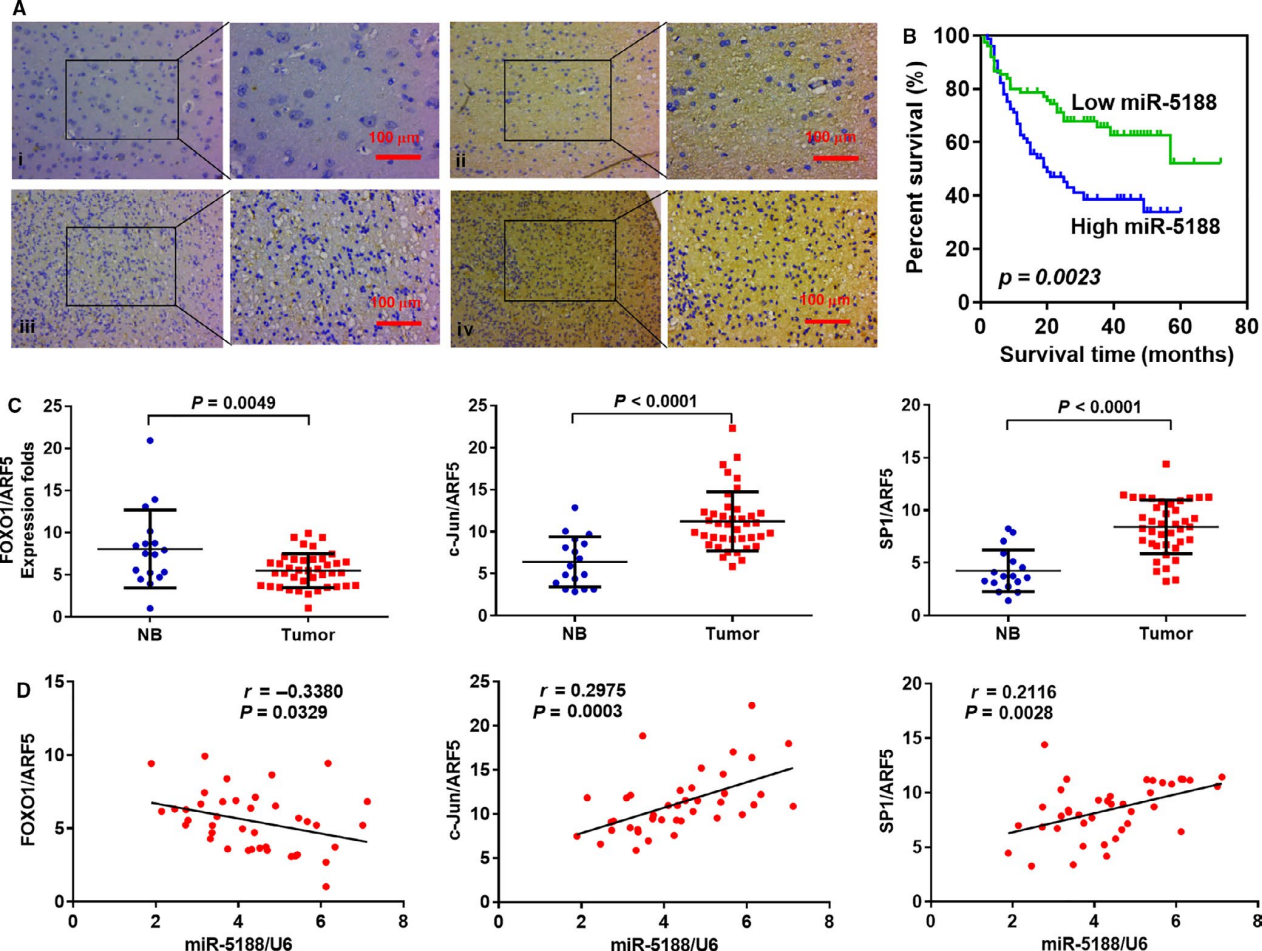
The correlations between pathoclinical characteristics, miR‐5188 expression and its associated genes. A, miR‐5188 expression was elevated in glioma in comparison with non‐tumour brain tissues. (i) Weak staining of miR‐5188 in non‐tumour brain tissues; (ii) strong staining of miR‐5188 in non‐tumour brain tissues; (iii) negative staining of miR‐5188 in glioma tissues; and (iv) strong staining of miR‐5188 in glioma tissues (insets showing miR‐5188 staining photographed under high magnification). Scale bars, 100 μm. B, Kaplan‐Meier survival analysis was applied to correlate miR‐5188 levels and overall survival in 148 glioma patients. C, The mRNA levels of FOXO1, c‐JUN and SP1 were measured through RT‐qPCR in glioma (n = 40) and non‐tumour (n = 17) brain tissues, normalized to ARF5. *P* = .0049; *P* < .0001; *P* < .0001, respectively. D, Correlations between miR‐5188, FOXO1, c‐JUN, and SP1 levels were explored in glioma tissues (n = 40). *P* = .0329; *P* = .0003; *P* = .0028, respectively

Compared to healthy brain tissues, the increased expression of miR‐5188 in glioma was confirmed by in situ hybridization assay (Figure 7A and Table S4) (*P* = .005). Table S5 presented the pathoclinical characteristics of the glioma patients. The patient population consisted of 88 males patients and 60 females patients aged from 3 to 70 years (37 ± 28.73 years). No significant correlation between miR‐5188 levels and patient age, sex, histologic type or tumour location was found in the 148 glioma patients. However, miR‐5188 expression was shown to be positively associated with WHO grade and necrosis type (Table S5) (*P* < .001 and *P* = .016, respectively). Besides, miR‐5188 expression was shown to be negatively correlated with a good prognosis of glioma patients (log‐rank test, Figure 7B).

miR‐5188, FOXO1, c‐JUN and SP1 expression were measured to illustrate their potential correlations in glioma and healthy brain tissues. c‐JUN and SP1 mRNA levels were elevated in glioma in comparison with those in healthy brain tissues (Figure 7C; *P* < .0001), whereas FOXO1 mRNA levels were down‐regulated in glioma (*P* = .0049). miR‐5188 levels were negatively associated with FOXO1 mRNA levels (Figure 7D; *r* = −0.3380, *P* = .0329), but positively correlated with c‐JUN mRNA levels (Figure 7D; *r* = 0.2975, *P* = .0003) and SP1 mRNA levels (Figure 7D; r = 0.2116, *P* = .0028) in the glioma specimens.

## 4 DISCUSSION

The biological function of miR‐5188 has not yet been documented in glioma. In this work, miR‐5188 was demonstrated as an important factor conferring poor patient prognosis and promoted cell proliferation, migration, invasion and G1/S cell cycle transition to facilitate glioma progression. This study demonstrates that miR‐5188 serves as a potential tumour promoter in glioma.

The FOXO1 transcription factor belongs to the Forkhead family and is known as a tumour suppressor that regulates the expression of a series of cancer‐associated genes, such as cyclin D1, IGFBP‐1, p130, p27, p21, cyclin D2, FasL and Bim.^27^, ^28^, ^29^, ^30^, ^31^, ^32^ FOXO1 participated in modulating of many biological processes, such as apoptosis, DNA damage repair, cell cycle arrest and/or oxidative stress resistance,^23^ and decreased FOXO1 mRNA levels were detected in breast carcinoma, prostate cancer, osteosarcoma, hepatocellular carcinoma and Ewing sarcoma.^24^, ^25^, ^26^, ^27^, ^28^, ^29^, ^30^, ^31^, ^32^, ^33^, ^34^, ^35^, ^36^, ^37^, ^38^ Numerous signalling pathways and molecules are related to glioma pathogenesis. The PI3K/Akt signalling pathway, which is commonly hyperactivated in almost all human cancers,^39^ controls several fundamental cellular processes, including cell proliferation, migration, angiogenesis and cell repair. The previous report showed that FOXO1 and PI3K/AKT signalling pathway forms a negative feedback circuit.^40^ In the current investigation, miR‐5188 was found to target the FOXO1 mRNA 3′UTR and inhibit its expression to augment cell proliferation, migration and invasion in glioma. Our study showed that FOXO1 inhibited cell growth and metastasis in glioma, which are consistent with those in the previous study.^9^ We also found that miR‐5188 promoted PI3K/AKT signalling and the protein levels of cell cycle factor c‐JUN and other PI3K/AKT downstream components. Mechanistically, miR‐5188 exerted its biological effects by directly targeting FOXO1 to activate PI3K/AKT/c‐JUN signalling. Previous studies also revealed that miR‐5188 promotes cancer progression through directly targeting FOXO1.^10^, ^11^ However, the signalling pathway regulated by miR‐5188 was different from that in our study, suggesting that miR‐5188 regulated cancer progression in a cell context‐specific and cell type‐specific manner.

The pro‐oncogene c‐JUN could regulate miRNA to augment glioma cell growth and metastasis.^41^ In this work, bioinformatics analyses revealed two c‐JUN‐binding sequences located in the promoter region of miR‐5188. Consistently, our study further confirmed the enhanced transcription of miR‐5188 regulated by c‐JUN in glioma progression. c‐JUN is known as a downstream effector involved in FOXO1‐PI3K/AKT signalling.^42^ In this work, c‐JUN‐augmented miR‐5188 transcription was facilitated by FOXO1‐PI3K‐AKT axis, suggesting the formation of miR‐5188‐FOXO1‐PI3K/AKT‐c‐JUN feedback circuit in glioma. SP1 is a well‐known transcription factor and was shown to regulate non‐coding RNA expression to promote glioma progression.^43^, ^44^ Here, we showed that SP1 enhanced miR‐5188 expression to promote glioma cell growth and metastasis. Sp1 could interact with c‐JUN to regulate gene transcription.^45^ In this study, we also demonstrated that miR‐5188 expression was augmented by the interaction between SP1 and c‐JUN. We assumed that SP1 might facilitate c‐JUN‐augmented miR‐5188 transcription. Intriguingly, SP1 stimulated miR‐5188‐FOXO1‐PI3K/AKT‐c‐JUN axis, thus promoting glioma development.

The differential analysis of miR‐5188 expression levels in glioma detected using in situ hybridization assay and the relationship between miR‐5188 and clinical characteristics confirmed the oncogenic role of miR‐5188 in glioma. Furthermore, the expression pattern of FOXO1, SP1 and c‐JUN in glioma samples and the correlations between FOXO1, c‐JUN, SP1 and miR‐5188 levels suggested an vital role of miR‐5188 in glioma development through FOXO1, c‐JUN and SP1 dysregulation.

Collectively, the current study suggests that miR‐5188 generates a positive feedback circuit through FOXO1/PI3K/AKT/c‐JUN signalling that is activated by SP1. This study sheds light on the function of miR‐5188 within intricate regulatory networks in glioma and lays a foundation for the potential application of miR‐5188 as a target for glioma treatment.

## CONFLICT OF INTEREST

The authors confirm that there are no conflicts of interest.

## AUTHOR CONTRIBUTION

**Renhui Yi:** Data curation (lead); Methodology (equal); Resources (lead); Writing‐original draft (equal); Writing‐review & editing (equal). **Shaochun Yang:** Methodology (equal); Writing‐original draft (equal); Writing‐review & editing (equal). **Xian Lin:** Methodology (equal); Writing‐original draft (equal); Writing‐review & editing (equal). **Liangying Zhong:** Data curation (supporting); Resources (supporting). **Yuanyuan Liao:** Data curation (supporting); Resources (supporting). **Zheng Hu:** Data curation (supporting); Resources (supporting). **Tengyue Huang:** Data curation (supporting); Resources (supporting). **Hao Long:** Data curation (supporting); Resources (supporting); Resources (supporting). **Jie Lin:** Data curation (supporting); Resources (supporting). **Zhiyong Wu:** Data curation (supporting); Resources (supporting). **Cheng Xie:** Formal analysis (lead); Software (lead). **Shengfeng Ding:** Formal analysis (supporting); Software (supporting). **Jie Luo:** Formal analysis (supporting); Software (supporting). **Qisheng Luo:** Conceptualization (equal); Project administration (equal); Supervision (equal). **Ye Song:** Conceptualization (equal); Funding acquisition (lead); Project administration (equal); Supervision (equal); Validation (equal).

## Supporting information

Supplementary Material

## Data Availability

The data that support the findings of this study are available from the corresponding author upon reasonable request.
