# Supplementary material for: miR‐5188 augments glioma growth, migration and invasion through an SP1‐modulated FOXO1‐PI3K/AKT‐c‐JUN‐positive feedback circuit
Source: J Cell Mol Med. 2020 Sep 9;24(20):11800–13. doi: 10.1111/jcmm.15794 (PMC7579714; doi:10.1111/jcmm.15794)
Supplement: Supplementary file 1 — Supplementary Material [file JCMM-24-11800-s001.doc]

| gene | No. |  | sequence |
| --- | --- | --- | --- |
| c-JUN | 1 | Sense | 5’ GGCACAGCUUAAACAGAAA dTdT 3’ |
|  |  | Antisense | 3’ dTdT CCGUGUCGAAUUUGUCUUU 5’ |
|  | 2 | Sense | 5’ CGCAGCAGUUGCAAACAUU dTdT 3’ |
|  |  | Antisense | 3’ dTdT GCGUCGUCAACGUUUGUAA 5’ |
| SP1 | 1 | Sense | 5’ CAGCGUUUCUGCAGCUACCUUGACU dTdT 3’ |
|  |  | Antisense | 3’ dTdT GUCGCAAAGACGUCGAUGGAACUGA 5’ |
|  | 2 | Sense | 5’ GACAGGUCAGUUGGCAGACUCUACA dTdT 3’ |
|  |  | Antisense | 3’ dTdT CUGUCCAGUCAACCGUCUGAGAUGU 5’ |
| FOXO1 | 1 | Sense | 5’CUGCAUCCAUGGACAACAA dTdT 3’ |
|  |  | Antisense | 3’ dTdTGACGUAGGUACCUGUUGUU 5’ |
|  | 2 | Sense | 5’ CCAGAUGCCUAUACAAACA dTdT 3’ |
|  |  | Antisense | 3’ dTdT GGUCUACGGAUAUGUUUGU 5’ |
| miR-5188  mimics | Sense | | 5’ AAUCGGACCCAUUUAAACCGGAG 3’ |
|  | Antisense | | 3’ UUAGCCUGGGUAAAUUUGGCCUC 5’ |
| Negative  control | Sense | | 5’ UUUGUACUACACAAAAGUACUG 3’ |
|  | Antisense | | 3’ AAACAUGAUGUGUUUUCAUGAC 5’ |
| miR-5188 inhibitor | | | 5’ CUCCGGUUUAAAUGGGUCCGAUU 3’ |
| Inhibitor negative control | | | 5’ CAGUACUUUUGUGUAGUACAAA 3’ |

**Table S1.** The sequences used in this study.

**Table S2. The primers used in this study.**

| Primers name | Sequence (5’-3’) | |
| --- | --- | --- |
| FOXO1 | Forward | AAGGCCATCGAGAGCTCGGC |
|  | Reverse | GCTCGGCTTCGGCTCTTAGCA |
| c-JUN | Forward | CTGCGTCTTAGGCTTCTCC |
|  | Reverse | CTCGCCCAAGTTCAACAA |
| miR-5188 | AATCGGACCCATTTAAACCGGAG | |
| U6 | CTCGCTTCGGCAGCACATATA | |
| c-JUN -A | Forward | AATCGCCGAGAAGGGAC |
|  | Reverse | TTTAGGACGGGACTTGGGT |
| c-JUN -B | Forward | GGCCAGAATTTAGCGGACAA |
|  | Reverse | TAGCTGAAGCTCCGGTTTT |
| ARF5 | Forward | ATCTGTTTCACAGTCTGGGAC |
|  | Reverse | CCTGCTTGTTGGCAAATACC |
| SP1 | Forward | TGGCAGCAGTACCAATGGC |
|  | Reverse | CCAGGTAGTCCTGTCAGAACTT |

**Table S3. A list of antibodies used for Western blot, IHC staining, CHIP.**

| Name of anti-body | Cat.No | Company | Mol weight | Dilution (WB/IHC/CHIP) |
| --- | --- | --- | --- | --- |
| c-JUN | 9165 | CST | 43,48 kDa | 1:1000 (WB); 1:50 (CHIP) |
| FOXO1 | ab52857 | abcam | 80 kDa | 1:250 (IHC) |
| FOXO1 | 2880 | CST | 78-82 kDa | 1:1000 (WB) |
| P-AKT | 4060 | CST | 60kDa | 1:1000 (WB) |
| P-PI3K | 4228 | CST | 85 kDa | 1:1000 (WB) |
| AKT | 4691 | CST | 60kDa | 1:1000 (WB) |
| CCND1 | ab134175 | abcam | 34 kDa | 1:1000 (WB) |
| PCNA | 10205-2-AP | PTG | 36-38 kDa | 1:30 (IHC) |
| N-cadherin | 4061 | CST | 140 kDa | 1:1000 (WB) |
| PI3K | 60225-1-Ig | PTG | 85 kDa | 1:500 (WB) |
| Ki67 | Ab16667 | abcam |  | 1:100 (IHC) |
| β-actin | sc-1616 | Santa | 43 kDa | 1:1000 (WB) |
| SP1 | 5931s | CST | 90 kDa | 1:1000 (WB);1:50 (CHIP) |
| CDK4 | 12790 | CST | 30 kDa | 1:1000 (WB) |
| Vimentin | 10366-1-AP | PTG | 54 kDa | 1:1000 (WB) |

**Table S4.** miR-5188 expression in glioma and non-tumor brain tissues

| **Group** | **miR-5188 expression** | | **Total** | **P value** |
| --- | --- | --- | --- | --- |
|  | **High** | **Low** |  |  |
| Glioma | 73 (49.3) | 75 (50.7) | 148 | **0.005**** |
| Non-tumor | 6 (20.7) | 23 (79.3) | 29 |
| **Total** | 79 | 98 | 177 |  |

Table S5. Correlations between miR-5188 expression and the pathoclinic parameters in glioma

| **Characteristics** | **n** | **miR-5188 expression(%)** | | **P** |
| --- | --- | --- | --- | --- |
|  |  | **High expression** | **Low expression** |  |
| Gender  Male  Female  Age  ≥50  <50  Histologic Type  Astrocytic tumors  Oligodendroglial tumors  Other  Tumor Location  Frontal  Temporal  parietal  Occipital  Cerebellum  Other  WHO Grade  I+ II  III+IV  Necrosis  Absent  Present | 88  60  34  114  101  17  30  44  27  19  10  10  38  63  85  57  91 | 45 (51.1)  28 (46.7)  19 (55.9)  54 (47.4)  54 (53.4)  9 (52.9)  10 (33.3)  20 (45.5)  16 (59.3)  11(57.9)  5 (50.0)  4 (40.0)  17 (44.7)  19 (30.2)  54 (63.5)  21 (36.8)  52 (57.1) | 43 (48.9)  32(53.3)  15 (44.1)  60 (52.6)  47 (46.5)  8 (47.1)  20 (66.7)  24 (54.5)  11 (40.7)  8 (42.1)  5 (50.0)  6 (60.0)  21 (55.3)  44 (69.8)  31 (36.5)  36 (63.2)  39 (42.9) | 0.593  0.383  0.146  0.768  **<0.001*****  **0.016*** |

**
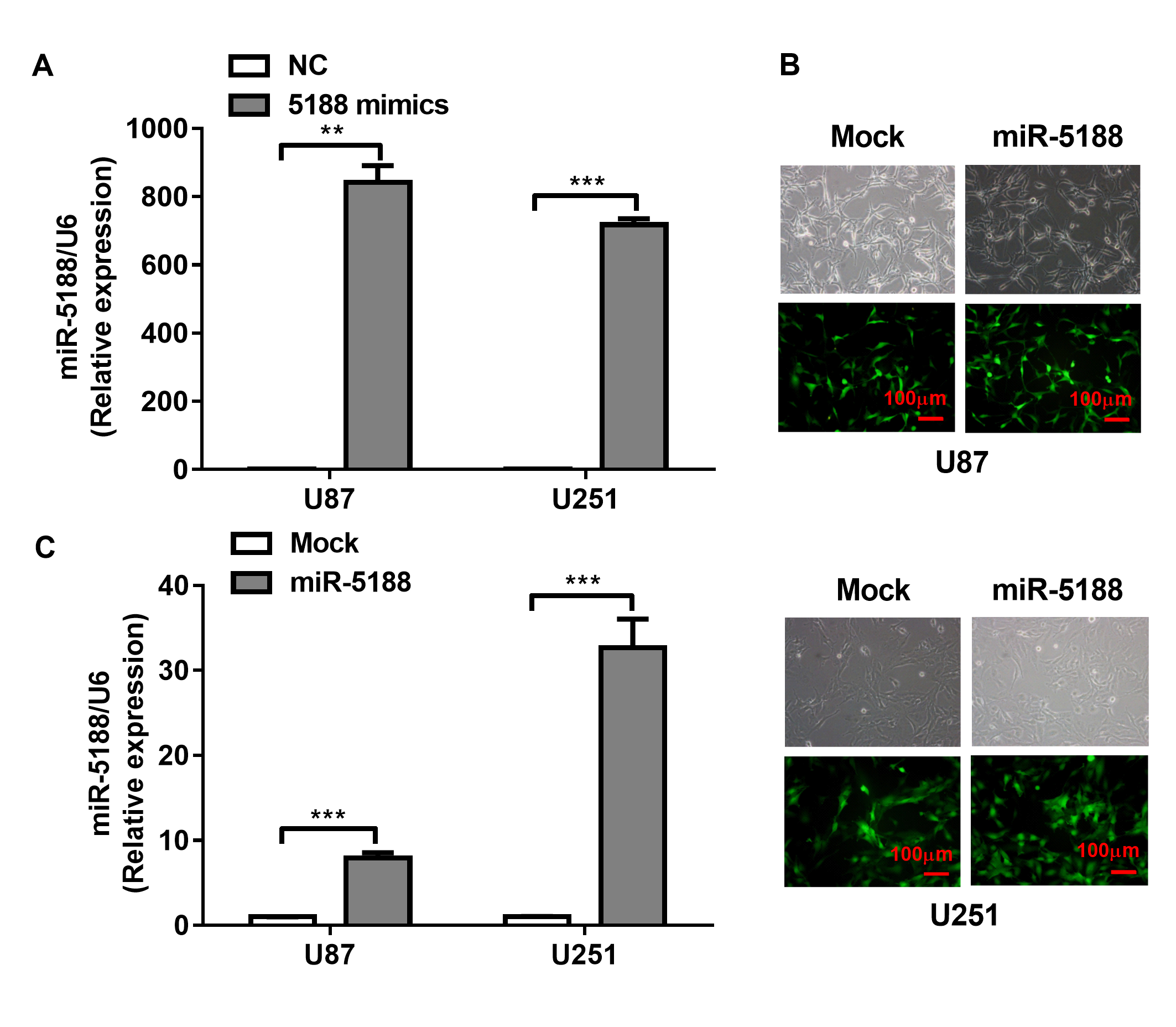
**

**Figure S1. A, C,** RT-qPCR showing transcriptional levels of the miR-5188 with U6 used as an internal control. The bar graph shows relative mRNA expression. Student’s t-test, mean ± SD, **P<0.01; ***P<0.001. **B,** U87 and U251 were transfected by lentiviruses containing miR-5188 overexpression (miR-5188) or untreated normal controls (mock).


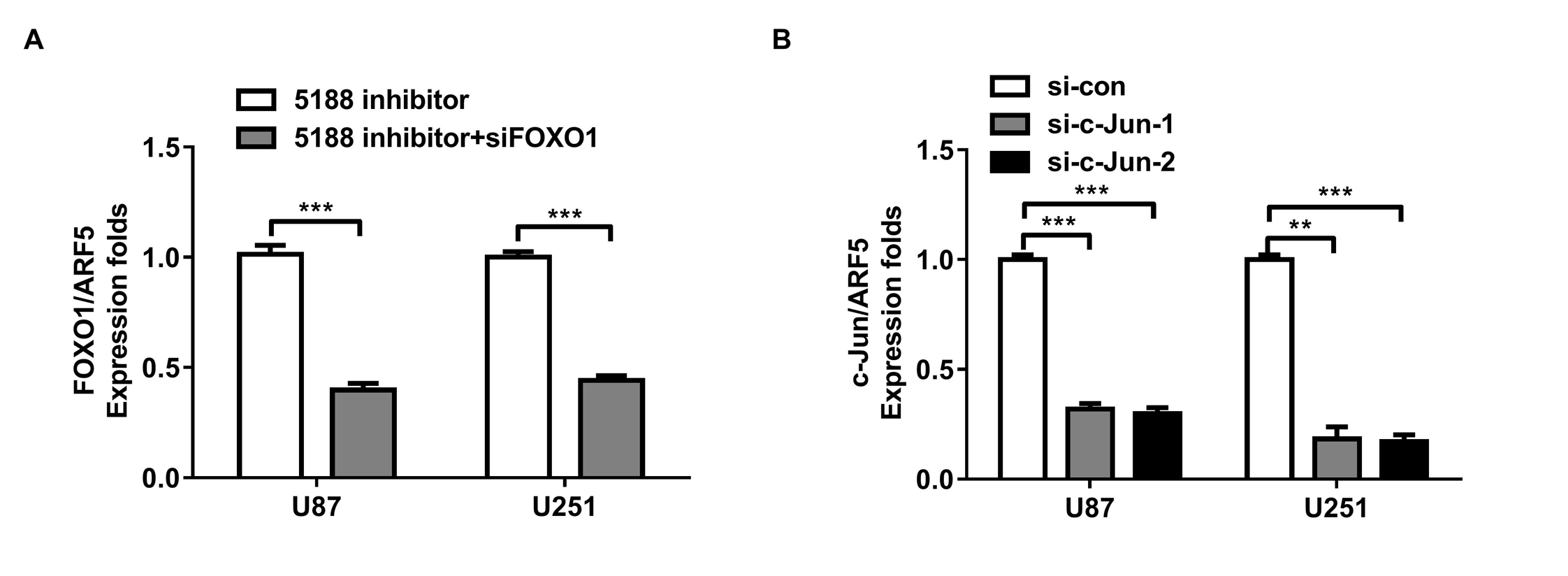


**Figure S2.** **A,** FOXO1 expression was detected by RT-qPCR in miR-5188-silenced or FOXO1-silenced U87 and U251 cells, normalized to ARF5. Student’s t-test. Mean ± SD. ***P<0.001. **B,** c-JUN expression was detected by RT-qPCR in c-Jun-suppressed U87 and U251 cells, normalized to ARF5. Student’s t-test. Mean ± SD, **P<0.01; ***P<0.001.
